# Full Polarization Conical Dispersion and Zero-Refractive-Index in Two-Dimensional Photonic Hypercrystals

**DOI:** 10.1038/srep22739

**Published:** 2016-03-09

**Authors:** Jia-Rong Wang, Xiao-Dong Chen, Fu-Li Zhao, Jian-Wen Dong

**Affiliations:** 1 School of Physics and State Key Laboratory of Optoelectronic Materials and Technologies, Sun Yat-sen University, Guangzhou, 510275, China

## Abstract

Photonic conical dispersion has been found in either transverse magnetic or transverse electric polarization, and the predominant zero-refractive-index behavior in a two-dimensional photonic crystal is polarization-dependent. Here, we show that two-dimensional photonic hypercrystals can be designed that exhibit polarization independent conical dispersion at the Brillouin zone center, as two sets of triply-degenerate point for each polarization are accidentally at the same Dirac frequency. Such photonic hypercrystals consist of periodic dielectric cylinders embedded in elliptic metamaterials, and can be viewed as full-polarized near zero-refractive-index materials around Dirac frequency by using average eigen-field evaluation. Numerical simulations including directional emissions and invisibility cloak are employed to further demonstrate the double-zero-index characteristics for both polarizations in the photonic hypercrystals.

Photonic Dirac cone has been discovered in two-dimensional photonic crystals of dielectric cylinders. The cone emerges at the corner of Brillouin zone, which is a counterpart to that in electronic solid state systems like graphene^1^,^2^,^3^ and topological insulators^4^,^5^,^6^. Such symmetry-protected cone plays an important role in the realization of nontrivial topological characteristics and non-zero Berry phase, inspiring many novel transport properties, such as photonic quantum Hall effect^7^ and photonic spin-momentum locking edge states^8^. On the other hand, a new kind of photonic Dirac cone at zone center has been reported in all-dielectric photonic crystals and aperiodic quasicrystals^9^,^10^,^11^,^12^,^13^,^14^. Different from those at zone boundary, the latter one is accidentally degenerate to form the conical dispersion with two linear bands and one flat band. Combined with effective medium theory, the triple-degenerate conical dispersion has an effective zero refractive index at Dirac frequency. Similar phenomena have been reported in two-dimensional phononic crystal^15^,^16^, possessing zero-index with simultaneous effective zero reciprocal of bulk modulus and zero mass density. In addition, the photonic Dirac cones are shown to exist in metamaterials as well^17^,^18^.

However, the cones are always dependent on polarizations of light in two-dimensional (2D) photonic crystals, which appears in either transverse magnetic (TM) or transverse electric (TE) polarization only. Polarization-independence, on the other hand, is highly expected in many research fields. For example, complete band gaps in both TM and TE polarizations are crucial in 2D and three dimensional (3D) photonic crystals^19^,^20^. Polarization-independent waveguides have been designed in 2D photonic crystal with full band gaps^21^,^22^, and in 3D photonic crystal with “accidental degeneracy” in guided dispersion relations for both polarizations by tuning line-defect profile^23^. Metamaterial also enables to manipulate polarizations. Polarization-independent negative refraction has been observed in “Swiss cross” structure in near infrared regime^24^. Wide-angle polarization-independent chiral metamaterial absorber has been also reported^25^. Recently, polarization-independent nontrivial band gap has been demonstrated in microwave regime by simultaneously breaking the four-fold degenerate Dirac points in the TE and TM polarizations with nonzero bianisotropy^26^,^27^.

In this paper, we demonstrate polarization-independent accidental Dirac cone in a class of photonic hypercrystal consisting of dielectric cylinders embedded in the elliptic metamaterial. The Dirac point is a six-fold accidentally degenerate state, derived from the overlapping of the two sets of Dirac frequencies of the TM and TE polarizations. By utilizing average eigen-field calculation, we demonstrate the full-polarized zero-refractive-index near the frequency of double Dirac cone. Directional emission and cloaking effect are simulated to illustrate the effective zero-refractive-index characteristics for both polarizations.

## Full polarization conical dispersion in photonic hypercrystals

### Triangular lattice with elliptic metamaterial background

**Figure 1:**
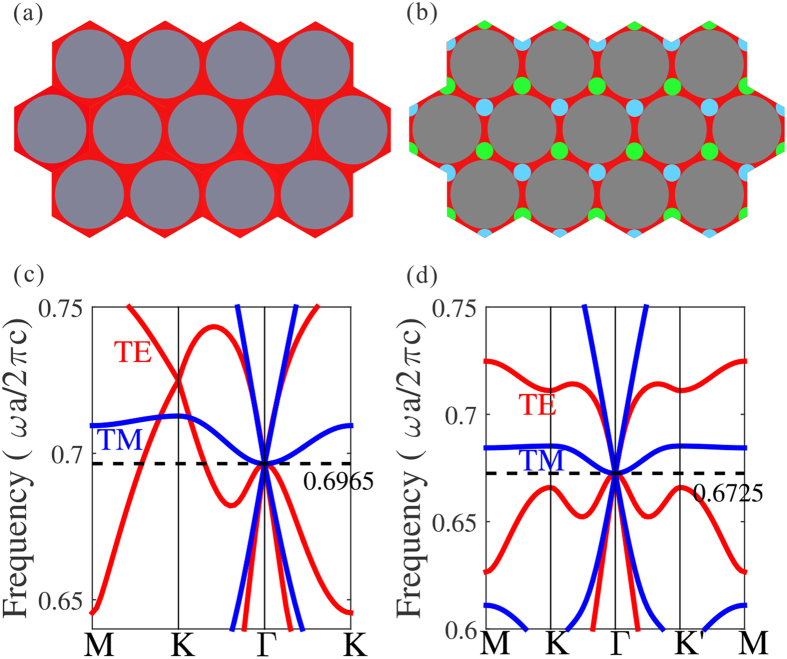
Full polarization conical dispersion in (**a,b**) triangular and (**c,d**) honeycomb photonic hypercrystals. (**a**) Schematic diagram of triangular photonic hypercrystal constructed by air holes in elliptic metamaterial of *ε*_host_ = (*ε*_//_, *ε*_//_, *ε*_⊥_), *ε*_//_ > 0 and *ε*_⊥_ > 0 (red). (**b**) Schematic diagram of honeycomb photonic hypercrystal with C_3v_ symmetry. The host (red) is made by elliptic metamaterial, while the hollow is air hole. Between holes, there are two sets of rods (green and blue) with different isotropic permittivity and same radii. (**c**) Band structure of the triangular photonic hypercrystal. Blue and red solid lines stand for transverse magnetic (TM) and transverse electric (TE) polarizations, respectively. The Dirac points for both polarizations are at the same frequency of 0.6965 *c/a*, forming the six-fold accidentally degeneracy at Γ point. (**d**) Band structure of the honeycomb photonic hypercrystal, showing the six-fold accidentally degenerate Dirac state at 0.6725 *c/a* and the lifted degeneracy at K point in TE polarization, which will benefit to improve the functionality of the zero-refractive-index photonic crystals.

It is well known that the condition on the accidentally degenerate Dirac cone in either TE or TM polarization is opposite. The TM-polarized Dirac cone prefers to the dielectric cylinders in air host, while the TE polarized Dirac cone prefers to the air rods embedded dielectric host. However, there is an exceptional case when the radii of the air rods are large enough in such way that each rod almost connects with each other. The large air-rod configuration effectively creates nearly “isolated” dielectric region so that it might support the TM-polarized Dirac cone. For example, consider a triangular lattice of air rods as shown in Fig. 1(a). The radius of the air rods is 0.4584*a*, where *a* is the lattice constant. The background medium (red) is a kind of elliptic metamaterial with the permittivity value of *ε*_host_ = (*ε*_//_, *ε*_//_, *ε*_⊥_) = (15.47, 15.47, 12.45). It can be constructed by layer-by-layer natural materials, so as to form the 2D photonic hypercrystal (more details later). Here, the use of anisotropy is to achieve two Dirac points of both polarizations at the same frequencies, as *ε*_//_ and *ε*_⊥_ can independently manipulate the appearance of accidentally degenerate Dirac cones for each polarization. Figure 1(c) shows the corresponding band structure of the triangular lattice with anisotropic metamaterial background. Blue lines refer to the TM polarization while red lines for the TE case. The conical dispersion and a triply accidental degenerate Dirac point appears at the center of Brillouin zone (Γ point) for both polarizations, with the frequency of *ωa/2πc* = 0.6965. In other words, there is a six-fold degenerate Dirac point. Detailed eigen-field pattern analysis shows that the eigen-state at the Dirac point consists of two monopolar singlets and two dipolar doublets, implying the feature of polarization independent zero refractive index (will be discussed later).

However, the TE-polarized band suffers from the high-k mode problem. This can be found in the red band of Fig. 1(c), that at the Dirac frequency, there are not only modes in small **k** near zone center, but also modes in large **k** near zone boundary due to double degeneracy protected by point group symmetry at K point. As a result, multimodes will be excited when placing a dipolar source inside the photonic hypercrystal, and it may seriously affect the predominant feature of the photonic device such as directional emitter.

### Honeycomb lattice of elliptic metamaterial background

**Figure 2:**
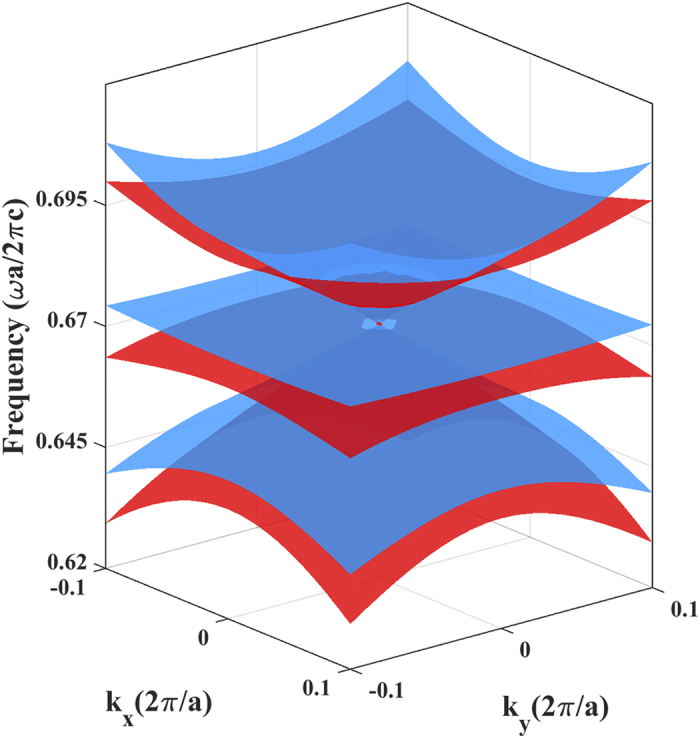
Three-dimensional dispersion surfaces near the double Dirac cones of the C_3v_ honeycomb photonic crystal. It shows the six-fold accidentally-degenerate conical dispersion for both polarizations. Red color represents the TE-polarized 3D surface and blue is TM-polarized.

The solution is to degrade the lattice symmetry in order for breaking the degeneracy at K point. The C_3v_ symmetry honeycomb structure is a good candidate, e.g., adding a set of small blue rods with different dielectric constant at the corner of the unit cell in Fig. 1(b). Another necessity to annihilate high-k mode issue is to form the frequency isolated point in such a way that the frequency of the flat band near K point are lower than the Dirac frequency at Γ point. This can be accomplished by increasing the severity of the broken degeneracy, e.g. adjusting the radii of small blue rod and big air rod. Note that the “isolated” region of the background should be kept and the dielectric constant of the background needs to be tuned to enable six-fold accidental degeneracy. The TE cone is determined by the in-plane component of dielectric constant *ε*_//_, while the TM case by the out-of-plane component *ε*_⊥_. But it does not guarantee the overlapping of the two Dirac frequencies. This is tricky to be solved when introducing a set of green rods at the other corner of the unit cell in Fig. 1(b). Followed the procedures above, a representative example is that the anisotropic permittivity of the host medium has the value of *ε*_*host*_ = (20.27, 20.27, 10.2) [red in Fig. 1(b)], while the isotropic blue and green rods at the corner is 12 and 21, respectively. The radii of the corner and the air rod are set to be 0.119*a* and 0.456*a*. Figure 1(d) shows the corresponding band structure. The TM-polarized Dirac cone overlaps with the TE-polarized cone at the frequency of 0.6725 *c/a*, exhibiting the conical dispersion and six-fold accidentally degeneracy. We also plot the three-dimensional dispersion surface of the band structure near the Dirac point in Fig. 2, in order to illustrate the Dirac cone. One can see that there are two conical cones touching at the Γ point with a flat sheet for both TM and TE polarizations. Note that the polarization-independent Dirac cone is sensitive to the values of permittivity and geometry parameters. The derivation of such parameters may lead to either the lift of double cones, or the frequency shift of the two Dirac points. The very-specific permittivity above is for illustrating the conical dispersion physics in a much more clear way. In realistic system, the specific permittivity is hard to satisfy, but it is possible to control the geometry parameters instead thanks to the nowadays nanoscale technologies.

### Proposal of elliptic metamaterial background

It is worthwhile to note that the anisotropic host in the unit cell of photonic hypercrystal is hard to be constructed by natural material. Composite metamaterial, on the other hand, offers us with a solution. Here, we first consider the multilayer model to build the anisotropic dielectric constant. The multilayer consists of two alternative dielectrics with high and low permittivity, and it is placed horizontally in the xy plane so that the effective permittivity can yield by the formulism^28^,^29^. The host material [*ε*_host_ = (15.47, 15.47, 12.45)] of the triangular photonic crystal in Fig. 1(a) can be approximately made by the high dielectric with the permittivity of 15.8 and the air layer when the filling ratio of high dielectric is 0.982. For the host material [*ε*_host_ = (20.27, 20.27, 10.2)] in the honeycomb photonic crystal in Fig. 1(b), it may be approximately realized when the filling ratio of the high dielectric is 0.947 and the two permittivities are 21.3 and 1.0. Such configuration may be achieved by low loss ceramic and foam in microwave experiment. Alternatively, the host material may be obtained by the structure of metal nanowire embedded dielectric matrix in optics experiment^29^. For example, according to Eqs (18) and (19) in ref. [29], we have the effective parameters of host material in Fig. 1(a), when the permittivity of dielectric and metal is 12.96 and −19 around λ = 0.62 μm, and the filling ratio of metal nanowire is 0.016. Another example is to set the permittivity of dielectric and metal to be 12.96 and −26 around λ = 0.72 μm with the metal filling ratio of 0.072, in order to obtain the effective parameters of host material in Fig. 1(b).

## Zero-refractive-index characteristics in photonic hypercrystals

### Retrieval of the effective zero-refractive-index

**Figure 3:**
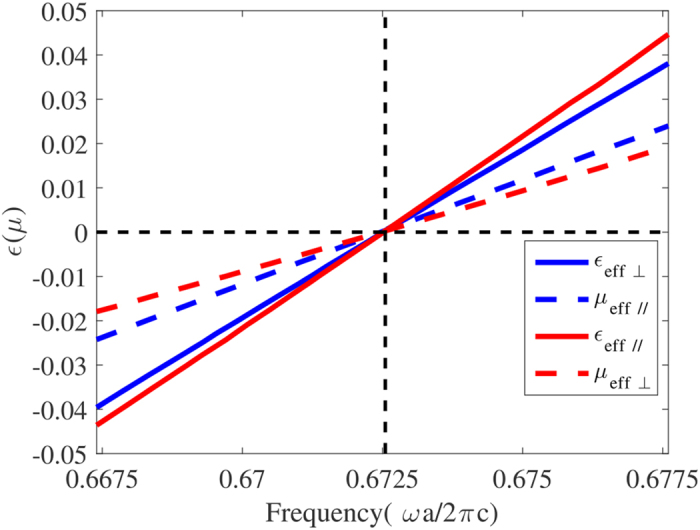
Effective medium parameters in the C_3v_ honeycomb photonic crystal by using average eigen-field method. All the components of effective permittivity and effective permeability go to zero for both polarizations, when the operating frequency approaches to the Dirac frequency. Note that the TM-polarized eigen-field is used to determine the out-of-plane component of the effective permittivity *ε*_*eff* ⊥_ (blue solid) and the in-plane components of the effective permeability *μ*_*eff* //_ (blue dash). On the other hand, the remaining effective parameters are retrieved from the TE-polarized eigen-field patterns, i.e., the in-plane effective permittivity *ε*_*eff* //_ (red solid) and the out-of-plane effective permeability *μ*_*eff* ⊥_ (red dash).

In this section, we will prove the existence of the effective zero-refractive-index near the Dirac frequency in the C_3v_ honeycomb photonic hypercrystal. The method here is the average eigen-field method^30^. Consider the light propagates along ΓM direction of the C_3v_ honeycomb lattice structure in Fig. 1(b). The effective parameters at a given frequency can be retrieved by


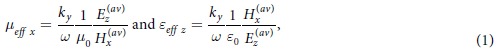


for the TM polarization, and


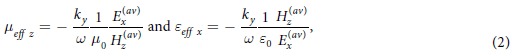


for the TE polarization. Here, 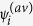 is the average eigen-field in the *i* direction, with the expression of 
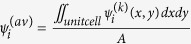
, where *ψ*_i_ = *E*_*i*_*,H*_*i*_, *i* = *x,z* and *A* is the area of the unit cell. To determine the *y* component of the effective parameters, we investigated the equifrequency contour and found that the contours in the frequency region near the double Dirac cone are almost circle, indicating that *n*_*eff y*_ ≈ *n*_*eff x*_ for both polarizations. Thus, we reach *μ*_*eff x*_ ≈ *μ*_*eff y*_* *=* μ*_*eff* //_ and *ε*_*eff x*_ ≈ *ε*_*eff y*_* *=* ε*_*eff* //_. Using the above method, the effective parameters of the C_3v_ honeycomb photonic crystal are retrieved from 0.6675 *c/a* to 0.6755 *c/a* and plotted in Fig. 3. All the components of the effective parameters exhibit quite a linear relation and have near-zero values in the vicinity of the Dirac frequency of 0.6725 *c/a*, confirming the polarization-independent effective zero-refractive-index.

### Directional emission

**Figure 4:**
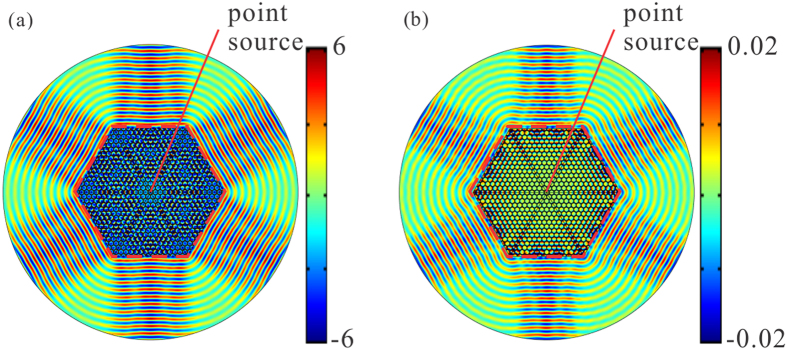
Dirac multidirectional emission in the honeycomb photonic hypercrystal. (**a**) TM-polarized near-field profiles from the hexagonal emitter constructed by honeycomb photonic hypercrystal. (**b**) TE-polarized near-field profiles from the same emitter. The photonic hypercrystal is the same as Fig. 1(b). Both cases are achieved at the Dirac frequency of 0.6725 *c/a*. The emitter device is illustrated in the red dash line with a point source at the center.

**Figure 5:**
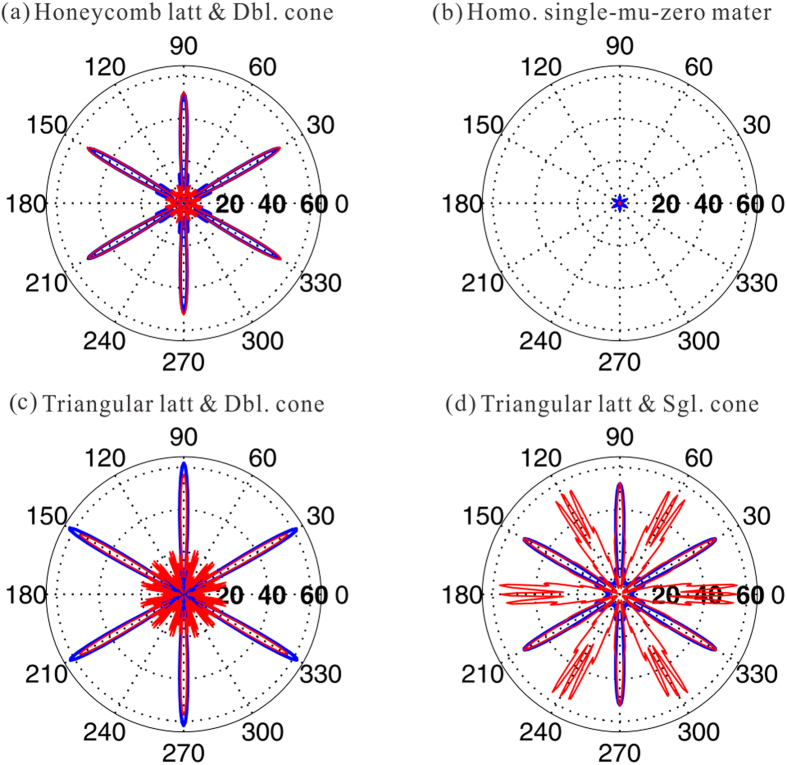
Dirac multidirectional emission in different kinds of zero-index photonic crystals/metamaterial. (**a**) Far-field profiles from the hexagonal emitter constructed by honeycomb photonic hypercrystal of Fig. 1(b). The polarization-independent directional emission is achieved at the Dirac frequency. Blue is for TM case while red for TE case. (**b**) Same as (**a**) except for the device of isotropic mu-near-zero materials with the values of *ε* = 1 and *μ* = 0.001. Little energy can emit outward due to impedance mismatch. (**c**) Same as (**a**) except for the device of the triangular lattice with double Dirac cone and high-k mode issue. The TE-polarized directivity is affected seriously. (**d**) Same as (**a**) except for the device of the triangular lattice, with rod radius of 0.184a and rod permittivity of 12.5. There is TM-polarized-only Dirac cone, and thus the TE-polarized direction emission fails.

As the effective zero-refractive-index at the Dirac points for both polarizations has been demonstrated in last section, directional emission, as one of the characteristic of zero-refractive-index, is expected in a six-port emitter of the photonic hypercrystal. To see this, we set a E_z_ polarized source at the Dirac frequency of 0.6725 *c/a* in the center of the finite hexagonal sample with the side length of 15*a*, whose schematic diagram is illustrated in Fig. 4(a). Propagating wave directionally emits along six directions out of the emitter, as demonstrated in the near field [Fig. 4(a)] and far field electric pattern [blue in Fig. 5(a)]. The pattern for the TE-polarized case is almost the same as the TM-polarized case when the H_z_ polarized source is employed [Fig. 4(b) and red in Fig. 5(a)]. In comparison, the emission in another six-port emitter made by single near-zero index material with the parameter of *ε* = 1, *μ* = 0.001 is plotted in Fig. 5(b). The far field pattern keeps the directionality but the emitted energy is seriously weaken due to impedance mismatch. To see the high-k mode issue, we employed the triangular photonic hypercrystal of Fig. 1(a) as example. Although the double Dirac cones are present for both polarizations, the TE-polarized wave is seriously affected [Fig. 5(c)]. Figure 5(d) gives another example with photonic crystal with single Dirac cone in TM polarization, reported in ref. [9], with rod radius of 0.184a, and rod permittivity of 12.5. Since TE-polarized Dirac cone does not exist in this structure, and the TE directionality is totally lost in Fig. 5(d). To conclude, the emitter with the honeycomb photonic hypercrystal can overcome various drawbacks above.

### Cloaking effect

**Figure 6:**
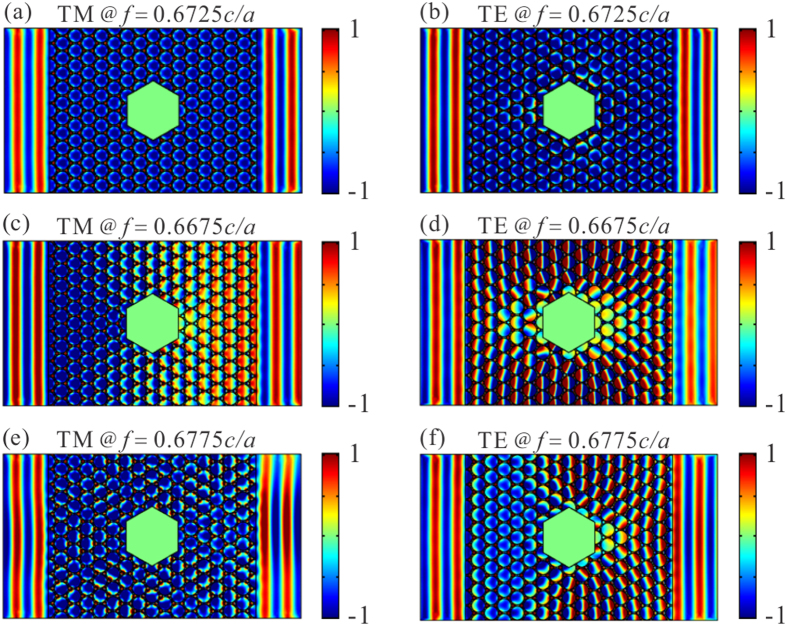
Cloaking effect for both polarizations in the honeycomb photonic hypercrystal near Dirac frequency for TM and TE polarizations. (**a**) E_z_ distributions when the waveguide boundary and the obstacle are perfect magnetic conductor at the Dirac frequency. The transmitted wave does not introduce additional phase change and it can maintain the plane wave profile without scattering and distortion. The phase change inside the photonic hypercrystal is almost the same due to the effective zero-refractive-index feature. (**c**)/(**e**) Same as (**a**) except the frequency is a little bit lower/higher than Dirac frequency when the effective-refractive-index of the photonic hypercrystal is around 0.03/−0.03 (~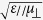). Similar cloaking effects still survive despite of obvious phase inside the crystal. (**b**) H_z_ patterns in the waveguide with perfect electric conductor boundary and obstacle inside at the Dirac frequency of 0.6725 *c/a*. (**d**)/(**f**) Same as (**b**) except the working frequency. Similar effects as those in TM polarization. Note that the cases in (**e**) and (**d**) refer to the excitation of high-k mode, see the band structure in Fig. 1(d).

Another interesting characteristic of zero-refractive-index is cloaking effect. Here, we calculated the transmission behaviors through a waveguide made of the honeycomb photonic hypercrystal with the propagating length of 8
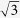
*a* and the height of 11*a*, when a hexagonal obstacle with the side length of 2*a* is placed inside the photonic hypercrystal waveguide. Note that the perfect magnetic conductor waveguide boundary and obstacle are used for TM polarization while the perfect electric conductor for TE polarization. In addition, two air rectangular area with the length of 3*a* is placed at both sides of the crystal waveguide to illustrate the profile of the wavefront. The E_z_ field patterns in Fig. 6(a) and the H_z_ field patterns in Fig. 6(b) show at the Dirac frequency, that the waveguide enables to preserve the transmitted wave as the plane wavefront without phase change, even when the obstacle is inserted. We also observe similar cloaking effect within a narrow bandwidth near the Dirac frequency as plotted in Fig. 6(c–f). The phase change is clearly seen inside the crystal as the effective refractive index is around 0.03(−0.03), but the output plane wavefront is preserved. Note that the validity of the bandwidth allows to relax the sensitivity of the specific values in permittivity and geometry parameters, even though the Dirac frequencies in two polarizations have a little deviation.

## Conclusions

In conclusion, we show the two-dimensional photonic hypercrystal exhibits polarization-independent conical dispersion and zero-refractive-index. The hypercrystal is made of a class of periodic lattice with elliptic metamaterial background that may be constructed by multilayer structure or nanowire structure. The conical dispersion locates at the same frequency for both polarizations, showing the frequency-isolated six-fold accidental degenerate point. By applying average eigen-field calculation, the full polarized zero-refractive-index is proved near Dirac frequency. Numerical simulations involving directional emission and cloaking effect are used to verify the effective zero-refractive-index characteristic for both polarizations.

## Additional Information

**How to cite this article**: Wang, J.-R. *et al.* Full Polarization Conical Dispersion and Zero-Refractive-Index in Two-Dimensional Photonic Hypercrystals. *Sci. Rep.* **6**, 22739; doi: 10.1038/srep22739 (2016).
